# Effectiveness of the trivalent influenza vaccine in Navarre, Spain, 2010–2011: a population-based test-negative case–control study

**DOI:** 10.1186/1471-2458-13-191

**Published:** 2013-03-06

**Authors:** Iván Martínez-Baz, Víctor Martínez-Artola, Gabriel Reina, Marcela Guevara, Manuel García Cenoz, Julio Morán, Fátima Irisarri, Maite Arriazu, Esther Albeniz, Jesús Castilla

**Affiliations:** 1 Instituto de Salud Pública de Navarra, Leyre 15, 31003, Pamplona, Spain; 2 CIBER Epidemiología y Salud Pública, Pamplona, Spain; 3 Complejo Hospitalario de Navarra, Pamplona, Spain; 4 Clínica Universidad de Navarra, Pamplona, Spain; 5 Dirección de Atención Primaria, Servicio Navarro de Salud, Pamplona, Spain

**Keywords:** Influenza A virus, H1N1 subtype, Influenza vaccines, Case–control studies, Spain

## Abstract

**Background:**

Some studies have evaluated vaccine effectiveness in preventing outpatient influenza while others have analysed its effectiveness in preventing hospitalizations. This study evaluates the effectiveness of the trivalent influenza vaccine in preventing outpatient illness and hospitalizations from laboratory-confirmed influenza in the 2010–2011 season.

**Methods:**

We conducted a nested case–control study in the population covered by the general practitioner sentinel network for influenza surveillance in Navarre, Spain. Patients with influenza-like illness in hospitals and primary health care were swabbed for influenza testing. Influenza vaccination status and other covariates were obtained from health care databases. Using logistic regression, the vaccination status of laboratory-confirmed influenza cases was compared with that of test-negative controls, adjusting for age, sex, comorbidity, outpatient visits in the previous 12 months, health care setting, time between symptom onset and swabbing, period and A(H1N1)pdm09 vaccination. Effectiveness was calculated as (1-odds ratio)x100.

**Results:**

The 303 confirmed influenza cases (88% for A(H1N1)pdm09 influenza) were compared with the 286 influenza test-negative controls. The percentage of persons vaccinated against influenza was 4.3% and 15.7%, respectively (p<0.001). The adjusted estimate of effectiveness was 67% (95% CI: 24%, 86%) for all patients and 64% (95% CI: 8%, 86%) in those with an indication for vaccination (persons age 60 or older or with major chronic conditions). Having received both the 2010–2011 seasonal influenza vaccine and the 2009–2010 pandemic influenza vaccine provided 87% protection (95% CI: 30%, 98%) as compared to those not vaccinated.

**Conclusion:**

The 2010–2011 seasonal influenza vaccine had a moderate protective effect in preventing laboratory-confirmed influenza.

## Background

Influenza is an important health problem that can lead to serious complications in persons with risk factors [1,2]. Annual vaccination is the primary measure for preventing influenza and its complications [3]. Because the influenza vaccine composition is adapted each season to the viruses in circulation, its effectiveness varies [4]. In the absence of clinical trials, observational studies are the main way to evaluate vaccine effectiveness in each season, however, a number of biases affecting comparability between vaccinated and unvaccinated persons must be overcome [5-8]. Studies looking at poorly specified outcomes tend to underestimate the effect of the intervention [6], whereas those that analyze virologically-confirmed cases reduce this problem [4,9]. A design that compares confirmed influenza cases with test-negative controls tends to improve the comparability and is easy to carry out, thus this type of study has come to be widely used [4,9-14]. Several studies have applied this methodology in patients recruited in primary care, which provides an estimate of the effect of the vaccine in preventing mild cases [9-12]. Other studies have analyzed hospitalized patients, which provides an estimate of vaccine effectiveness in preventing serious forms of illness [13,14].

This study uses a test-negative case–control design nested in a population-based cohort to evaluate the effectiveness of the influenza vaccine in the 2010–2011 season in preventing laboratory-confirmed influenza, including both outpatient and hospitalized patients.

## Methods

### Study population

The present study was based on electronic clinical records in the region of Navarre, Spain. The Navarre Ethical Committee for Medical Research approved the study protocol.

The Navarre Health Service provides health care, free at point of service, to 97% of the population of the region. The clinical records have been computerized since 2000, and include those from primary care, hospital admissions and laboratory test results.

In Navarre the trivalent inactivated non-adjuvanted vaccine was recommended and offered free of charge to people aged 60 or over and to those with major chronic conditions. Other people can also be vaccinated if they pay for the vaccine. In the 2010–2011 season the vaccine included strains A/California/07/2009(H1N1)-like, A/Perth/16/2009(H3N2)-like and B/Brisbane/60/2008-like virus [15]. Monovalent influenza A(H1N1)2009 vaccine [16] had been administered to individuals with major chronic conditions in the 2009–2010 season. Precise instructions for registering each dose were given to all vaccination points [17].

Influenza surveillance was based on automatic reporting from the electronic medical records of all cases of influenza-like illness (ILI) from all primary health care centers and hospitals. In both settings ILI was considered to be the sudden onset of any general symptom (fever or feverishness, malaise, headache or myalgia) in addition to any respiratory symptom (cough, sore throat or shortness of breath). A sentinel network of 74 primary care physicians and pediatricians were asked to take swabs, after obtaining verbal informed consent, from all their patients diagnosed with ILI whose symptoms had begun preferably less than 5 days previously. An agreed protocol of care for influenza cases was applied in hospitals, which specified early detection and nasopharyngeal swabbing of all hospitalized patients with ILI. Swabs were processed by real-time reverse transcription polymerase chain reaction (RT-PCR) assay in two laboratories in the region, and positive samples were characterized for influenza A (H1 and H3), A(H1N1)pdm09 and B virus. Two positive swabs for each week were sent to the national influenza reference laboratory for genotyping.

### Study design and statistical analysis

We carried out a nested case–control study based on electronic clinical records. The study population included all persons over 6 months of age who received medical care from physicians in the sentinel network. Health care workers and institutionalized persons were excluded. The study population included 97,597 persons. The study began the first week in which influenza virus was detected following the first 15 days of the vaccination campaign and ended the week preceding two consecutive weeks in which none of the ILI patients recruited tested positive for influenza. Accordingly, the study covered the period from week 43 of 2010 to week 12 of 2011.

The cases were all patients diagnosed with influenza syndrome in primary care or in hospitals who were confirmed for influenza virus. The controls were patients with influenza syndrome who were negative for influenza virus.

The vaccination status for the trivalent 2010–2011 seasonal influenza vaccine and monovalent A(H1N1)pdm09 influenza vaccine was obtained from the online regional vaccination register [18]. Subjects were considered to be protected 14 days after vaccine administration.

From the electronic health care records we obtained the following baseline characteristics: sex, age, district of residence, major chronic conditions and primary health care visits in the previous 12 months. Logistic regression techniques were used to calculate crude odds ratios (ORs), and ORs adjusted for the mentioned variables, for health care setting, for swabbing within 4 days of symptom onset and for date of visit grouped into 4-week periods. When necessary, exact logistic regression was used.

The effects of the 2010–2011 seasonal vaccine and the monovalent A(H1N1)pdm09 vaccine were evaluated as independent variables in one model, and as a combined variable (unvaccinated, only seasonal vaccine, only pandemic vaccine, or both vaccines) in a different model.

Specific analyses were made under different situations: comparing cases of each type of influenza with negative controls, including only patients in whom influenza vaccination was indicated because they were 60 years of age or older or had some major chronic condition, considering only patients in primary care or only hospitalized patients, and including only swabs taken in the first 4 days after symptom onset. All adjusted analyses included both influenza vaccines, except the analysis of influenza B, since the pandemic vaccine did not include this virus. All relevant covariates were maintained in the models for each specific analysis. Percentages were compared by χ^2^. Vaccine effectiveness was estimated as (1-OR)x100.

## Results

### Description of cases and controls

**Figure 1:**
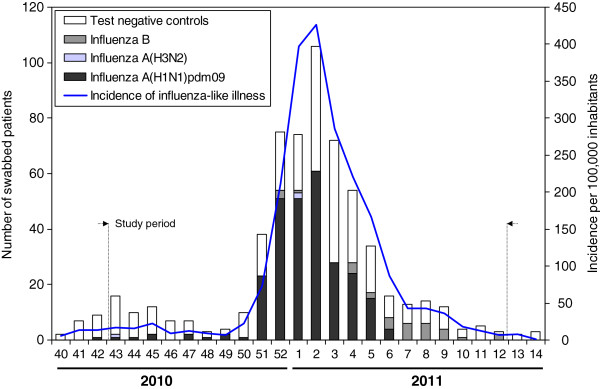
Number of test-negative controls and influenza cases, and incidence of influenza-like illness by week.

The weekly number of swabbed patients followed the pattern of ILI incidence in the population (Figure 1). During the study period, 589 ILI patients were swabbed, 530 in primary health care and 59 in hospitals. Some 51.4% (303) were confirmed for influenza virus: 267 for influenza A(H1N1)pdm2009, 3 for influenza A(H3N2) and 33 for influenza B. Thirty-one of these strains were characterized in the national influenza reference laboratory: 23 were A/California/07/2009(H1N1), 1 was A/England/142/2010(H1N1), 2 were A/HongKong/2121/2010(H3N2), 1 was B/Bangladesh/3333/2007(Yamagata) and 4 were B/Brisbane/60/2008(Victoria).

Compared with confirmed cases of influenza, test-negative controls had a higher proportion of persons who were age 65 or over, who had consulted a physician five or more times in the last year, who had more than one major chronic condition, who were diagnosed between week 51 of 2010 and 2 of 2011, and who were treated in the hospital. A smaller proportion of cases than controls had been vaccinated against influenza A(H1N1)pdm2009, and this was also true for the 2010–2011 seasonal vaccine. There were no differences between cases and controls by sex, residence or swabbing within 4 days of symptom onset. All patients were swabbed within 8 days of symptom onset (Table 1).

**Table 1:** Characteristics of cases and controls

	**Laboratory confirmed influenza cases**	**Test-negative controls**	
	**n (%)**	**n (%)**	**p-value**
**Total**	303 (100)	286 (100)	
**Age groups (years)**			0.004
< 5	10 (3.3)	9 (3.1)	
5 - 14	38 (12.4)	19 (6.6)	
15 - 44	174 (57.4)	151 (52.8)	
45 - 64	65 (21.5)	69 (24.1)	
65 - 79	13 (4.3)	26 (9.1)	
≥ 80	3 (1.0)	12 (4.2)	
**Sex**			0.387
Male	146 (48.2)	148 (51.7)	
Female	157 (51.8)	138 (48.3)	
**Residence**			0.943
Rural	43 (14.2)	40 (14.0)	
Urban	260 (85.9)	246 (86.0)	
**Major chronic conditions**			0.005
0	232 (76.5)	199 (69.6)	
1	59 (19.5)	56 (19.6)	
> 1	12 (4.0)	31 (10.8)	
**Outpatient visits in the previous year**			0.047
0	53 (17.5)	33 (11.5)	
1 to 4	134 (44.2)	120 (42.0)	
> 4	116 (38.3)	133 (46.5)	
**Health care setting**			<0.001
Primary health care	290 (95.7)	240 (83.9)	
Hospital	13 (4.3)	46 (16.1)	
**Period**			<0.001
Week 43 to 46/2010	5 (1.7)	40 (14.0)	
Week 47 to 50/2010	6 (1.9)	18 (6.3)	
Week 51/2010 to 2/2011	192 (63.4)	101 (35.3)	
Week 3 to 6/2011	81 (26.7)	95 (33.2)	
Week 7 to 12/2011	19 (6.3)	32 (11.2)	
**Delay between symptom onset and swabbing**			0.118
≤4 days	297 (98.0)	274 (95.8)	
>4 days	6 (2.0)	12 (4.2)	
**Pandemic influenza vaccine 2009-2010**			0.001
No	297 (98.0)	260 (90.9)	
Yes	6 (2.0)	26 (9.1)	
**Seasonal influenza vaccine 2010-2011**			<0.001
No	290 (95.7)	241 (84.3)	
Yes	13 (4.3)	45 (15.7)	

### Effectiveness of the 2010–2011 seasonal influenza vaccine

Compared with test-negative controls, a smaller proportion of confirmed influenza cases had received the 2010–2011 seasonal influenza vaccine (OR: 0.24; 95% CI: 0.13; 0.46; p<0.001). In the analysis adjusted for age, sex, major chronic conditions, outpatient visits in the previous year, swabbing within 4 days of symptom onset, health care setting, period and previous monovalent pandemic vaccination, the effectiveness of the seasonal vaccine was 67% (95% CI: 24%, 86%; p=0.009). In the analysis restricted to swabs taken within the first 4 days after symptom onset, the estimate was 65% (95% CI: 16%, 85%; p=0.018).

The effectiveness of the 2010–2011 influenza vaccine against cases of influenza A(H1N1)pdm2009 was 61% (95% CI: 9%, 83%; p=0.030). Using exact logistic regression techniques, influenza vaccine effectiveness against influenza B virus was estimated as 93% (95% CI: 36%, 100%; p=0.017).

In the analysis restricted to persons in whom the vaccine was indicated, the effectiveness was 64% (95% CI: 8%, 86%; p=0.034).

In the analysis that included only patients diagnosed in primary care, vaccine effectiveness rose to 68% (95% CI: 17%, 87%; p=0.019). Estimates of the vaccine effectiveness were similar for people aged less than 50 (73%; 95% CI: -21%, 94%; p=0.086) and those aged 50 or over (69%; 95% CI: 0%, 91%; p=0.050) (Table 2).

**Table 2:** Influenza vaccine effectiveness in preventing laboratory-confirmed influenza in the 2010–2011 season in Navarre, Spain

	**Cases/controls**	**Crude vaccine effectiveness, % (95% CI)**	**p-value**	**Adjusted vaccine effectiveness, % (95% CI)**	**p-value**
**All swabbed patients**					
Pandemic vaccine 2009-2010					
No	297/260	Ref.		Ref.	
Yes	6/26	80 (50; 92)	<0.001	45 (−68; 82) ^**a**^	0.297
Seasonal vaccine 2010-2011					
No	290/241	Ref.		Ref.	
Yes	13/45	76 (54; 87)	<0.001	67 (24; 86) ^**a**^	0.009
**Patients swabbed within 4 days of symptom onset**					
Pandemic vaccine 2009-2010					
No	292/250	Ref.		Ref.	
Yes	6/25	79 (49; 92)	0.001	44 (−71; 82) ^**a**^	0.309
Seasonal vaccine 2010-2011					
No	286/235	Ref.		Ref.	
Yes	12/40	75 (52; 87)	<0.001	65 (16; 85) ^**a**^	0.018
**Influenza A(H1N1)pdm09 cases vs. controls**					
Pandemic vaccine 2009-2010					
No	262/260	Ref.		Ref.	
Yes	5/26	81 (49; 93)	<0.001	60 (−30; 87) ^**a**^	0.129
Seasonal vaccine 2010-2011					
No	254/241	Ref.		Ref.	
Yes	13/45	73 (48; 86)	<0.001	61 (9; 83) ^**a**^	0.030
**Influenza B cases vs. controls**					
Seasonal vaccine 2010-2011					
No	33/241	Ref.		Ref.	
Yes	0/45	89 (33; 100)^c^	0.010	93 (36; 100) ^c,d^	0.017
**Subjects with indication for seasonal vaccination **^**b**^					
Pandemic vaccine 2009-2010					
No	82/85	Ref.		Ref.	
Yes	6/21	70 (24; 89)	0.013	28 (−147; 79) ^**a**^	0.599
Seasonal vaccine 2010-2011					
No	76/69	Ref.		Ref.	
Yes	12/37	71 (39; 86)	0.001	64 (8; 86) ^**a**^	0.034
**Primary health care patients**					
Pandemic vaccine 2009-2010					
No	286/227	Ref.		Ref.	
Yes	4/13	76 (24; 92)	0.015	56 (−70; 89) ^**e**^	0.231
Seasonal vaccine 2010-2011					
No	280/216	Ref.		Ref.	
Yes	10/24	68 (31; 75)	0.003	68 (17; 87) ^**e**^	0.019
**Hospitalized patients**					
Pandemic vaccine 2009-2010					
No	11/33	Ref.		Ref.	
Yes	2/13	54 (−137; 91) ^c^	0.355	8 (−967; 94) ^c,e^	1.000
Seasonal vaccine 2010-2011					
No	10/25	Ref.		Ref.	
Yes	3/21	64 (−47; 91) ^c^	0.154	61 (−316; 98) ^c,e^	0.677
**Subjects aged less than 50 years**					
Pandemic vaccine 2009-2010					
No	246/191	Ref.		Ref.	
Yes	2/6	74 (−30; 95)	0.100	60 (−65; 94) ^**a**^	0.341
Seasonal vaccine 2010-2011					
No	245/188	Ref.		Ref.	
Yes	3/9	75 (4; 93)	0.043	73 (−21; 94) ^**a**^	0.086
**Subjects aged 50 years or older**					
Pandemic vaccine 2009-2010					
No	51/69	Ref.		Ref.	
Yes	4/20	73 (26; 91)	0.024	47 (−133; 88) ^**a**^	0.401
Seasonal vaccine 2010-2011					
No	45/53	Ref.		Ref.	
Yes	10/36	67 (27; 85)	0.007	69 (0; 91) ^**a**^	0.050

*CI* confidence interval.

^**a**^ Estimates obtained by a logistic regression model including 2010–2011 seasonal and monovalent A(H1N1)pdm09 influenza vaccination, and adjusted for sex, age, major chronic conditions, outpatient visits in the previous year, swabbing within 4 days of symptom onset, health care setting and period.

^b^ Indication for vaccination includes people age ≥60 years old and people with major chronic conditions.

^c^ Estimates obtained by exact logistic regression.

^d^ Estimates obtained by a logistic regression model including 2010–2011 seasonal influenza vaccination, and adjusted for sex, age, major chronic conditions, outpatient visits in the previous year, swabbing within 4 days of symptom onset, health care setting and period.

^e^ Estimates obtained by a logistic regression model including 2010–2011 seasonal and monovalent A(H1N1)pdm09 influenza vaccination, and adjusted for sex, age, major chronic conditions, outpatient visits in the previous year, swabbing within 4 days of symptom onset and period.

### Effect of the monovalent A(H1N1)pdm09 vaccine received in the previous season

A smaller proportion of influenza cases than controls had received the pandemic vaccine (2.0% and 9.1%, respectively; p=0.001). However, in the multivariate analyses adjusted for the main covariates and for 2010–2011 seasonal vaccination, the protective effect of the pandemic vaccine was partially diluted and did not reach statistical significance (Table 2).

Table 3 shows the specific effectiveness of both vaccines as a combined variable (unvaccinated, only seasonal vaccine, only pandemic vaccine, or both vaccines). In persons who had not received the pandemic vaccine, the effect of the 2010–2011 seasonal vaccine in preventing confirmed influenza cases was 64% (95% CI: 14%, 85%; p=0.022), whereas having received both vaccines provided 87% (95% CI: 30%, 98%; p=0.017) protection against laboratory-confirmed influenza in the 2010–2011 season. The interaction term between the two vaccines was not statistically significant (p=0.502). Estimates of vaccine effectiveness were similar both in the analysis restricted to cases of influenza A(H1N1)pdm2009 and in the analysis of the target population for vaccination.

**Table 3:** Estimates of the effect of the 2010–2011 seasonal influenza vaccine and monovalent influenza A(H1N1)pdm09 vaccine in preventing laboratory-confirmed influenza, Navarre, Spain 2010–2011

	**Case/control**	**Crude vaccine effectiveness, % (95% CI) **^**a**^	**P-value**	**Adjusted vaccine effectiveness, % (95% CI) **^**b**^	**P-value**
**All swabbed patients**					
Seasonal and pandemic vaccines	2 / 19	91 (63; 98)	0.001	87 (30; 98)	0.017
Only seasonal vaccine	11 / 26	65 (28; 83)	0.004	64 (14; 85)	0.022
Only pandemic vaccine	4 / 7	53 (−62; 86)	0.230	21 (−252; 82)	0.755
Unvaccinated	286 / 234	Ref.		Ref.	
**Influenza A(H1N1)pdm09 cases vs. controls**					
Seasonal and pandemic vaccines	2 / 19	91 (57; 98)	0.002	86 (25; 97)	0.022
Only seasonal vaccine	11 / 26	61 (18; 81)	0.012	60 (2; 83)	0.044
Only pandemic vaccine	3 / 7	60 (−56; 90)	0.187	53 (−132; 91)	0.351
Unvaccinated	251 / 234	Ref.		Ref.	
**Subjects with indication for seasonal vaccination**					
Seasonal and pandemic vaccines	2 / 16	89 (50; 97)	0.004	81 (−9; 97)	0.062
Only seasonal vaccine	10 / 21	58 (3; 81)	0.041	50 (−38; 82)	0.179
Only pandemic vaccine	4 / 5	29 (−176; 82)	0.623	−32 (−694; 78)	0.763
Unvaccinated	72 / 64	Ref.		Ref.	

^a^ Logistic regression model including 2010–2011 seasonal and monovalent A(H1N1)pdm09 influenza vaccination status as a combined variable (unvaccinated, only seasonal vaccine, only pandemic vaccine, or both vaccines).

^b^ Logistic regression model including 2010–2011 seasonal and monovalent A(H1N1)pdm09 influenza vaccination status as a combined variable (unvaccinated, only seasonal vaccine, only pandemic vaccine, or both vaccines) and adjusted for sex, age, major chronic conditions, outpatient visits in the previous year, swabbing within 4 days of symptom onset, health care setting and period.

## Discussion

The results of this study show a moderate protective effect of the 2010–2011 seasonal influenza vaccine in preventing laboratory-confirmed influenza during the 2010–2011 seasonal period in Navarre, during which there was a good match between the circulating viruses and those included in the vaccine. The vaccine was shown to be effective against the two main viruses circulating in the region during the season: A(H1N1)pdm09 virus and B virus. Unlike other studies, ours included both outpatient and hospital cases systematically recruited in a previously defined population.

The effectiveness we found is similar or slightly higher than that described in other studies made in outpatients in the 2010–2011 season, with small differences that could be explained by the pattern of viruses circulating in each place [10,11].

The results concerning a possible residual effect of monovalent influenza A(H1N1)pdm09 vaccination in the previous season were not conclusive. As in other studies, a greater protective effect was observed with both vaccines than with seasonal vaccine alone [11,12], but in our study this difference was not statistically significant. Influenza A(H1N1)pdm09 virus was found in 88% of the laboratory-confirmed influenza cases and was included in both vaccines. Since this was a new virus with which most of the population had had no previous contact, dual vaccination may have helped to achieve a better immune response [19-21]. However, this finding could be due to biases since previous receipt of the pandemic vaccine may be an indicator of health-seeking behaviours.

The seasonal influenza vaccine had a high effectiveness against influenza B. Most characterizations of influenza B viruses were similar to B/Brisbane/60/2008(Victoria), which was the strain included in the seasonal vaccine [15].

Longer time between symptom onset and swabbing has been associated with reduced sensitivity in virus detection, which could underestimate vaccine effectiveness [6]. We controlled for this effect mainly in the design of our study, since 97% of the swabs were taken within the first 4 days after symptom onset. Moreover, all analyses were adjusted for this variable and the analysis was repeated after eliminating the cases swabbed after the first 4 days, with no relevant changes found in the estimate of vaccine effectiveness.

In Navarre, the vaccine was indicated for all persons aged 60 or over and for persons with major chronic diseases that increase the risk of influenza complications. Restricting the analysis to this population group, the effectiveness of the influenza vaccine was similar, despite the fact that factors such as advanced age or some immunodepression may be more common among people with major chronic conditions, which would explain poor response to the vaccine.

Primary care patients made up the bulk of subjects in our study and, when the analysis was limited to these patients, the effectiveness of the vaccine was maintained. The number of cases treated in hospitals was small, which did not allow us to obtain a statistically significant estimate of the effect of the vaccine in preventing hospitalized cases. Nevertheless, a study conducted in the same region and season to evaluate vaccine effectiveness in preventing hospitalizations with confirmed influenza in a risk population found a 59% vaccine effectiveness [14].

This case–control analysis included only laboratory-confirmed cases and compared them with test-negative controls recruited in the same health care settings before either patient or physician knew the laboratory result, a fact that provides better comparability between cases and controls and reduces selection bias [6]. This type of design has been used in other studies that have evaluated influenza vaccine effectiveness [10-14]. The case–control study was nested in a population-based cohort for which extensive and reliable databases are available, and which is treated by sentinel physicians trained to detect and swab ILI patients, all of which helps to prevent unmeasured confounding [22]. All the analyses were adjusted for the effect of the most commonly recognized confounding factors.

## Conclusion

These results support a moderate protective effect of the 2010–2011 seasonal vaccine and do not rule out a possible low residual effect of the monovalent pandemic vaccine against influenza in the 2010–2011 season. Our findings can be added to those of other studies that highlight the importance of annual immunization of high-risk populations against influenza, complemented with other preventive initiatives such as promotion of basic hygiene measures and avoiding contact with influenza cases.

## Abbreviations

ILI: Influenza-like illness; CI: Confidence interval; OR: Odds ratio.

## Competing interests

The authors declare that they have no competing interests.

## Authors’ contributions

IMB and JC designed the study, data analysis, interpreted the results and wrote the first draft of the article. MG participated in data analysis and interpretation. VMA, GR, MGC, JM, EA, FI, MA collected data and participated in the interpretation of the results. All authors revised and approved the final draft. IMB and JC are equally responsible for this article.

## Pre-publication history

The pre-publication history for this paper can be accessed here:

http://www.biomedcentral.com/1471-2458/13/191/prepub
